# Vitamin D Pathway Genes, Diet, and Risk of Renal Cell Carcinoma

**DOI:** 10.1155/2010/879362

**Published:** 2009-11-05

**Authors:** S. Karami, P. Brennan, M. Navratilova, D. Mates, D. Zaridze, V. Janout, H. Kollarova, V. Bencko, V. Matveev, N. Szesznia-Dabrowska, I. Holcatova, M. Yeager, S. Chanock, N. Rothman, P. Boffetta, W-H. Chow, L. E. Moore

**Affiliations:** ^1^Division of Cancer Epidemiology and Genetics, National Cancer Institute, NIH, DHHS, Bethesda, MD, USA; ^2^International Agency for Research on Cancer, Lyon, France; ^3^Department of Cancer Epidemiology and Genetics, Masaryk Memorial Cancer Institute, Brno, Czech Republic; ^4^Institue of Public Health, Bucharest, Romania; ^5^Institute of Carcinogenesis, Cancer Research Centre, Moscow, Russia; ^6^Department of Preventive Medicine, Faculty of Medicine, Palacky University, Olomouc, Czech Republic; ^7^Institute of Hygiene and Epidemiology, First Faculty of Medicine, Charles University in Prague, Czech Republic; ^8^Department of Epidemiology, Institute of Occupational Medicine, Lodz, Poland; ^9^Core Genotyping Facility, Advanced Technology Center, Bethseda National Cancer Institute, NIH, Department of Health and Human Services, MD, USA

## Abstract

Mediated by binding to the high-affinity vitamin D receptor (VDR), vitamin D forms a heterodimer complex with the retinoid-X-receptor (RXR). Variation in both genes has been shown to modify renal cell carcinoma (RCC) risk. Therefore, we investigated whether *VDR* and *RXRA* polymorphisms modify associations between RCC risk and frequency of dietary intake of vitamin D and calcium rich foods, and occupational ultraviolet exposure among 777 RCC case and 1035 controls from Central and Eastern Europe. A positive association was observed in this population between increasing dietary intake frequency of yogurt, while an inverse association was observed with egg intake frequency. *RXRA* polymorphisms, located 3′ of the coding sequence, modified associations between specific vitamin D rich foods and RCC risk, while *RXRA* polymorphisms, located in introns 1 and 4, modified associations with specific calcium rich foods. Results suggest that variants in the *RXRA* gene modified the associations observed between RCC risk and calcium and vitamin D intake.

## 1. Introduction

Smoking, obesity, and hypertension are well-established risk factors of renal cell carcinoma (RCC) [1, 2]. Other risk factors such as dietary practices have been implicated, though inconsistent results have usually been reported for specific dietary components [1, 3]. Diets rich in fruits and vegetables however have typically been shown to decrease RCC risk possibly through antioxidant effects, while consumption of fried foods has commonly been shown to increase risk possibly due to the potential carcinogenic effects of acrylamides [1–3]. Recently, epidemiological studies suggest that vitamin D, which is found in food (vitamin D_2_ and D_3_) and produced in the body after exposure to ultraviolet (UV) rays from the sun (vitamin D_3_), may be inversely associated with RCC risk [4–7]. 

Both vitamin D_2_ and D_3_ are hydroxylated in the liver and subsequently in the kidney to form active vitamin D (1,25(OH)_2_D_3_) [8]. Dietary intake of vitamin D accounts for a small (approximately 10%) proportion of vitamin D levels [8–10]. However, dietary intake of vitamin D and vitamin D rich foods may play a role in determining RCC risk since vitamin D has been associated with anticarcinogenic properties and is primarily metabolized within the kidneys [8, 11]. 

Although the exact anticarcinogenic mechanism of vitamin D is not fully understood, vitamin D and its metabolites are thought to impede carcinogenesis by stimulating cell differentiation, inhibiting cell proliferation, inducing apoptosis, and suppressing invasiveness, angiogenesis, and metastasis [11–14]. Vitamin D is mediated by binding to vitamin D receptors (*VDRs*), transcription factors that are part of the nuclear hormone receptor family. Forming a heterodimer complex with the retinoid-X-receptor (*RXR*) gene, *VDR* can regulate the transcription of other genes involved in cell regulation, growth, and immunity [13, 15, 16]. Most epidemiological studies have generally focused on the *VDR* gene, however recently, we evaluated 139 single nucleotide polymorphisms (SNPs) across eight genes of the vitamin D pathway and found a significant association between RCC risk and across certain *VDR* and *RXRA* genetic variants [17]. 

Given that the kidney is the most important organ for vitamin D metabolism and activity and calcium homeostasis and that within the kidneys, calcium has been shown to influence active vitamin D levels [8, 17], investigation of dietary vitamin D and calcium in RCC etiology are highly relevant. We therefore investigated whether common genetic variation in *VDR* and *RXRA*, the two genes we observed to be significantly associated with RCC risk, modified associations between renal cancer risk and the frequency of dietary intake of vitamin D and calcium rich foods. Additionally, we also explored whether these genes modified occupational UV exposure and RCC risk, since recently we reported a significant inverse association between occupational UV exposure and RCC risk among male participants in this population [18] and because the majority of people worldwide obtain most of their vitamin D levels from the sun [8–10]. Analyses were conducted among cases and controls from Central and Eastern Europe, an area with one of the highest rates of RCC worldwide and an area where foods are not regularly fortified with either vitamin D or calcium [19]. 

## 2. Materials and Methods

### 2.1. Study Population

Details regarding this study population were previously described [20]. Briefly, from 1999 through 2003, a hospital-based case-control study of RCC was conducted in seven centers in four countries of Central and Eastern Europe (Moscow, Russia; Bucharest, Romania; Lodz, Poland; Prague, Olomouc, Ceske-Budejovice, and Brno, Czech Republic). Cases, 20 to 88 years of age, included newly diagnosed patients (within 3 months) with histologically confirmed RCC (IDC-O-2 codes C64) who resided in the study areas for at least one year. Controls in all centers were frequency matched to cases on age (±3 years), sex, and place of residence. Controls were chosen among subjects admitted as inpatients or out patients in the same hospital as the cases, with non-tobacco-related conditions, including infections (1.1%), hematologic (3.2%), endocrine (2.0%), psychiatric (1.4%), neurologic (11.2%), ophthalmologic or otologic (14.5%), cardiovascular (9.6%), pulmonary (3.9%), gastrointestinal (18.7%), dermatologic (2.8%), orthopedic or rheumatologic (8.9%), genitourinary (benign prostatic hyperplasia) (3.8%), obstetric or perinatal (0.1%), injury or poisoning (3.0%), and other (15.9%). No single disease made up more than 20% of the control group. Furthermore, a number of controls were also recruited in parallel for studies of lung and head and neck cancers [21, 22]. A total of 1097 incident, histologically confirmed RCC cases and 1476 controls were included in this study. Suitable quantity and quality of genomic DNA was obtained from a subset of 777 (70.8%) eligible RCC cases and 1035 (70.1%) controls enrolled in our study. The response rates across study centers for study participation ranged from 90.0% to 98.6% for cases and from 90.3% to 96.1% for controls. All subjects provided written informed consent. This study was approved by the institutional review boards of all participating centers.

### 2.2. Dietary Intake

Details of dietary assessment have been previously described [23]. Briefly, interviewers were trained in each center to perform face-to-face interviews with cases and controls during hospitalization using standard questionnaires. The questionnaire covered demographic characteristics, family history of cancer, history of tobacco consumption, and dietary habits. A food frequency questionnaire was comprised of 23 food items, which the study investigators selected by consensus during the planning stage of the study and further validated during the pilot stage by asking participants to name common food items not already specified [23]. Frequency of dietary intake for each food group was assessed for each item as never, less than once per month, less than once per week, one to two times per week, three to five times per week, and daily. A standardized questionnaire was used in each of the study centers that was translated from a common English version and then backtranslated into English to ensure the validity of the initial translation. The questionnaire was repeated for two different time periods: (1) the year prior to interview, and (2) the year prior to political and market changes in 1989 (1991 in Russia). Data for the year prior to interview and the year prior to political change were then extrapolated to represent lifetime average dietary intake by multiplying the score for each time period by the number of years the participant was alive during the time period, then summing the time period scores and dividing by the total age of the individual as previously described.

In this study, intake frequencies of vitamin D rich foods which include liver, eggs, and fish were combined for each participant to create a new dietary exposure variable for total vitamin D intake frequency. Since calcium has been reported to influence vitamin D levels and risk of certain cancers [8], intake frequencies of cheese, milk, and yogurt were also combined for each participant to create a new total calcium intake frequency exposure variable. In this study, specific details regarding the type of fish, cheese, milk, and so forth consumed was not available; therefore, each food category was assumed to have equal weights when combined to estimate total vitamin D or total calcium lifetime average dietary intake frequency. Very few foods naturally contain vitamin D and in the United States (US) and Canada, fortified foods are common sources of vitamin D [8, 9]. However, in most countries, such as those in Central and Eastern Europe, foods are not fortified with vitamin D or calcium. Therefore the major sources of vitamin D intake are fatty fish, such as mackerel and salmon, while the main source of calcium intake is primarily dairy products [7–9]. All dietary intake frequencies in this study were assessed in tertiles based on the frequency of dietary intake among controls.

### 2.3. Occupational UV Exposure

Lifetime occupational information for jobs held for at least 12 months duration was also ascertained during interviews through the use of a general occupational questionnaire. Data collected for each job included title, detailed tasks, and type of employer as well as the year of beginning and ending employment. These data were used to create a job-exposure matrix (JEM) which classified each study subjects' estimated occupational exposure to sunlight; the JEM is described in more detail elsewhere [18]. To assess the relationship between estimated occupational sunlight exposure, RCC risk and variation in vitamin D pathway genes, cumulative exposure across all jobs (calculated as duration (years) **x** frequency midpoint **x** intensity of exposure for each job and summed over all jobs held) was assessed. Since the association between occupational UV exposure and RCC risk in this population was previously observed only among males [18], evaluation of genetic variants, occupational UV exposure, and RCC risk are only presented for male participants. The relationship between these exposures and RCC risk was also explored among female participants; however no association was observed (data not shown). A categorical exposure matrix was used to evaluate the association between RCC risk and cumulative occupational UV exposure based on tertiles of exposure levels among male controls. 

### 2.4. Laboratory Procedures

Genomic DNA was extracted from whole blood buffy coat using a standard phenol chloroform method at the National Cancer Institute (NCI) laboratory. Genotyping was conducted with a GoldenGate Oligo Pool All (OPA) assay by Illumina (www.illumina.com). DNA samples from RCC cases and controls were coded and randomized on polymerase chain reaction (PCR) plates for genotyping analyses. A random 5% duplicate sample was selected and dispensed across plates genotyped for quality control. 

As previously reported in this population [17], the ten SNPS across the *VDR* gene (rs11574027, rs4760648, rs2853564, rs2254210, and rs886441) and *RXRA* gene (rs100791, rs3118523, rs748964, rs3118536, and rs10776909) that were significantly associated with RCC risk were further evaluated in this study to see if SNPs modified associations between RCC risk and dietary or occupational UV exposure. Tag SNPs were originally selected to provide 80% to 90% coverage across the genomic regions of interest and were applicable, while nonsynonymous SNPs were selected for their putative functional significance. All SNPs selected for analysis had a variant allele frequency of at least 5% as reported by the NCI SNP500Cancer Database (http://snp500cancer.nci.nih.gov) [24] and had a validated assay at the NCI Core Genotyping Facility (CGF) (http://cgf.nci.nih.gov/home.cfm). The concordance rate between duplicate DNA samples ranged from 93% to 100% and completion rates ranged from 98% to 100%. The genotype frequencies among controls did not differ from the expected Hardy-Weinberg equilibrium proportions (*P* > .05). 

### 2.5. Statistical Analysis

The distributions of select characteristics and known RCC risk factors (sex, age, smoking habits, self-reported hypertension status, body mass index (BMI), family history of cancer, country of residence, and particular dietary variables) were compared between cases and controls using the Chi-square test. Odds ratios (ORs) and 95% confidence intervals (95% CIs) using unconditional logistic regression were calculated to estimate the associations between individual SNPs and RCC risk.

SNP variants were assessed using a dominant model using the Wald chi-square test for the presence or absence of the variant allele (0, 1). Occupational UV exposure was assessed in tertiles calculated from the exposure prevalence among male controls. All regression models were adjusted for age, sex, study center, smoking status (ever, never), BMI, and self-reported hypertensive status.

*VDR* and *RXRA* haplotype blocks identified previously [17] were analyzed in relation to RCC risk using Haplostats (R version 2.4.0; http://www.r-project.org) [25]. Associations between common haplotypes (>5% frequency) and RCC risk were evaluated by computing ORs and 95% CIs using the most common haplotype as the referent category. Interactions were tested by comparing regression models with and without interaction terms using a likelihood ratio test (LRT). Interactions between dietary components, select *VDR* and *RXRA* SNPs, and RCC risk were only assessed for total calcium, total vitamin D, and for intake frequency food groups that were found to be statistically associated with RCC risk (i.e., eggs and yogurt). 

All analyses were conducted in STATA 9.0 unless otherwise specified (STATA Corporation, College Station, TX). 

## 3. Results

Table 1 provides a description of genotyped study participants and known RCC risk factors. Cases and controls were comparable in age and sex; however, cases were more likely to have excess BMI (>30 kg/m^2^), self-reported hypertension, and have a first degree relative with cancer. After adjustment for age, BMI, hypertension, study center, and sex the association with smoking was no longer observed [26].

Associations between intake frequency of vitamin D and calcium rich foods and RCC risk among genotyped participants are shown in Table 2. A statistically significant reduction in RCC risk was observed with increasing consumption frequency of eggs (*P*-trend = .02). A borderline statistically significant reduction in RCC risk was observed with increasing consumption frequency of total vitamin D (*P*-trend = .09). In contrast, increased RCC risk was observed with increasing consumption frequency of yogurt (*P*-trend = .04). 

Associations between RCC risk, *RXRA* SNPs, and intake frequency of eggs and total vitamin D are shown in Table 3. Inverse associations were observed between RCC risk and subjects with the wild type rs1007971 (*P*-trend = .05), rs3118523 (*P*-trend = .01), rs748964 (*P*-trend = .01), rs3118536 (*P*-trend = .02), and rs10776909 (*P*-trend = .05) *RXRA* genotypes compared to individuals with at least 1 variant allele. However, after stratification by egg intake, reduced RCC risk was observed with increasing consumption frequency of eggs among subjects with ≥1 variant rs1007971 (*P*-trend <.001), rs3118523 (*P*-trend <.001), rs748964 (*P*-trend <.001), rs3118536 (*P*-trend <.001), and rs10776909 (*P*-trend = .004) allele across the *RXRA* gene. Interactions were only significant for *RXRA *SNPs located downstream, 3′ of the coding sequence that is, rs1007971 (*P*-interaction = .01), rs3118523 (*P*-interaction = .01), and rs748964 (*P*-interaction = .01). Although not statistically significant, a weaker inverse association between RCC risk and total vitamin D (based on liver, eggs, and fish) intake frequency was observed. No association was observed between any of the *VDR* SNPs and dietary consumption frequency of eggs or total vitamin D (data not shown). 

Associations between RCC risk, *RXRA* SNPs, and intake frequency of yogurt and total calcium are shown in Table 4. A positive association was observed between RCC risk and increasing consumption frequency of yogurt among participants with the wild type *CC* rs3118538 (*P*-trend = .01) and *CC* rs10776909 (*P*-trend = .01) alleles across the *RXRA* gene; rs10776909 located in intron 1 was also shown to significantly modify associations between frequency of dietary intake of yogurt and RCC risk (*P*-interaction = .04). Similarly, increased RCC risk was observed with increasing total calcium intake frequency for subjects with the wild type *CC* rs3118538 (*P*-trend = .05) and *CC* rs10776909 (*P*-trend = .02) alleles. Significant interactions were observed for both SNPs, located in introns 1 and 4, within the *RXRA* gene. No association was observed between intake frequency of yogurt or total calcium and the other SNPs (N = 8) previously shown to be associated with RCC risk in this study [17]. 

Previously we observed a significant reduction (38%) in RCC risk among male participants occupational exposed to UV [18]. In this study, neither *VDR* nor *RXRA* SNPs modified the observed associations (data not shown); however, certain *VDR* haplotypes, previously associated with RCC risk in this study [18], were found to modify associations between RCC risk and occupational UV exposure among male participants (Table 5). Males with the *VDR A-G-C* (rs2254210, rs2853564, and rs4760648) haplotype were at a significantly increased (OR = 1.27; 95%CI = 1.03–1.57) risk of renal cancer compared to males with the most common referent haplotype *G-A-T*. When haplotypes were stratified by occupational UV category, only males in the highest UV exposure category with the same *VDR* haplotype, *A-G-C*, were shown to have a significantly stronger increase in RCC risk (OR = 1.70; 95%CI = 1.14–2.53) compared to males with the referent haplotype, *G-A-T*. No associations between *VDR* haplotypes, RCC risk and cumulative occupational UV exposure were observed among male participants in the lowest or middle UV category. A borderline significant interaction was also observed between *VDR* haplotypes, occupational UV exposure, and RCC risk (*P*-interaction = .06).

## 4. Discussion

In this study, reduced RCC risk was observed with increasing consumption frequency of eggs while increased RCC risk was observed with increasing consumption frequency of yogurt. After stratification by *VDR* and *RXRA* genotypes, the two genes previously associated with RCC risk in this study [17], only *RXRA* gene variants modified associations between RCC risk and consumption frequency of eggs, yogurt, and total calcium. *RXRA* SNPs 3′ of the coding sequence modified the association for intake frequency of eggs while intronic *RXRA* SNPS modified the association for intake frequency of yogurt and total calcium. No associations with *VDR* or *RXRA* genotypes were observed with occupational UV exposure and RCC risk among male participants previously observed to have lower RCC risk with increasing occupational sunlight exposure [18]; however, when *VDR* haplotypes were examined male subjects in the highest cumulative occupational UV exposure category with the *A-G-C VDR* haplotype had a significant increase in RCC risk compared to male subjects with the referent *G-A-T* haplotype. 

Numerous studies have established a link between diet and cancer risk or progression, therefore the World Cancer Research Fund has recently acknowledged that after smoking, diet may be the second most important contributor to the global burden of cancer [27]. While, dietary vitamin D intake constitutes a small proportion of total vitamin D requirements [8, 9], studies suggest that dietary intake of vitamin D may play an important role in determining cancer risk [5, 28]. This is particularly important in areas where ultraviolet levels of exposure vary seasonally. Overall, most epidemiological studies investigating the association between RCC risk and vitamin D or calcium rich foods have been inconsistent and generally null [23, 28–32]. Similar to the results we present in this study, an Italian case-control study of 767 RCC cases and 1534 controls observed a significant inverse association between dietary vitamin D intake, based on a 78-item food frequency questionnaire, and RCC risk [5]. However, an earlier Canadian RCC case-control study showed no association for intake of individual foods rich in vitamin D (i.e., fish and eggs) or calcium (i.e., milk, diary, and cheese) [28]. In contrast, calcium supplement intake in that study was shown to significantly reduce RCC risk with increasing the number of years of intake [28]. 

While no reports to date have investigated the association between RCC risk, dietary intake of vitamin D or calcium rich foods, and vitamin D gene variants, results have been reported for *VDR* SNPs and other cancers. For example, two colorectal case-control studies, one in the US [33] and one in UK [34], observed reduced cancer risk with increasing intake of vitamin D and calcium rich foods among participants with the homozygous *VDR* variant (*polyA*, *BsmI*, and/or *ApaI*) alleles compared to participants with the wild type alleles. In contrast, a US case-control study reported increased colorectal cancer risk with increasing vitamin D intake for subjects with the homozygous variant *VDR* (*BsmI BB*) allele [35]. No association between vitamin D intake, *VDR* polymorphisms (*TaqI*, *BsmI*, *FokI*, *ApaI*, and/or *polyA*) and lymphoma [36] and breast cancer risk [37], was reported in two separate US studies.

Epidemiological studies investigating whether polymorphisms in vitamin D pathway genes modify the relationship between cancer risk and UV exposure have been inconsistent. Similar to our results, an association between UV exposure and *VDR* haplotypes comprised of the rs2254210 SNP was reported in a recent British prostate cancer case-control study. Four *VDR* SNPs in two haplotype block regions were shown to significantly increase cancer risk among men with low UV exposure [38]. However, inverse associations were reported in this population when five other *VDR* SNPs were examined in relation to UV exposure and prostate cancer risk [39]. Likewise, in a US case-control study of prostate cancer, reduced cancer risk was observed among subjects with the *FokI* and *TaqI FFtt* haplotype among subjects in the highest UV exposure category [40]. 

Our study adds to the limited epidemiological evidence that calcium and vitamin D (from dietary intake or UV exposure), and *VDR* and *RXRA* vitamin D pathway genes may play a role in RCC etiology. The biological activity of vitamin D is mediated by a high-affinity receptor, *VDR,* which acts as a ligand-activated transcription factor that forms a heterodimer with *RXR* [13, 15, 16, 41]. This *VDR-RXR* heterodimer complex is directed to the vitamin D-responsive element in the promoter region of 1,25-regulated genes [13, 15, 16, 41]. Different polymorphisms in the *VDR* gene have been speculated to result in variation of *VDR* expression and result in changes to circulating levels of active vitamin D [4]. For this reason, epidemiological studies suggest that tissue specific expression of vitamin D pathway genes function as the primary mechanism involved in linking vitamin D status with anticarcinogenic effects of 1,25(OH)_2_D_3_ [42]; therefore, lower renal cancer risk may be associated with higher circulating levels of 25(OH)D, the storage form of vitamin D by providing substrate for renal tissue-specific synthesis of 1,25(OH)_2_D_3_ [43]. *RXRA* on the other hand may play a critical role in vitamin D activity, particularly from dietary sources, since this gene has been shown to regulate cholesterol [44], which is abundant in eggs and yogurt, the food groups found statistically associated with renal cancer risk in this study. *RXRA* regulated fatty acid and cholesterol metabolism through intestinal cholesterol absorption and bile acid synthesis [44]. Cholesterol metabolism has been associated with arthrosclerosis which is associated with hypertension and cardiovascular risk, known risk factors of RCC [45]. 

Strengths of our study include the use of HapMap to tag genes of interest using high (80–90%) genomic coverage both 5′ and 3′ of the target genes. This study also observed high participation rates among newly diagnosed histologically confirmed cases and collected biologic materials from a high proportion of subjects. The large sample size of this study provided sufficient statistical power to detect relatively small associations; however, the power to detect gene-environment interactions was limited and results need to be interpreted cautiously. 

Inherent limitations of our study include ( 1) the possibility of nondifferential dietary recall bias, ( 2) possible misclassification of vitamin D and calcium intake, ( 3) the inability to control for potential confounders such as total caloric intake, which may bias results away from the null, ( 4) lack of data regarding dietary vitamin D or calcium supplements, and ( 5) limited statistical power for gene-environment interactions. Additionally, 18.7% of controls were selected among patients admitted with gastrointestinal conditions, which may potentially influenced dietary habits thus, affecting study results. Limitations stemming from the estimate of occupational UV exposure [18], such as nondifferential, inaccurate, or incomplete recall of all occupational histories may also have biased results. While hospital-based case-control studies have potential limitations due to possible differences with population controls, these studies can improve response rates for the intense collection of biological specimens and therefore reduce the chances of bias in the assessment of gene-environment interactions [46]. Although there is general agreement that the serum 25(OH) vitamin D level is the best indicator of current vitamin D status, the biochemical marker has a short half-life and a single measurement of 25(OH) vitamin D may not reflect long-term status that would be important in studies of cancer. Moreover, vitamin D status postdiagnosis among cases may reflect disease rather than historical vitamin D levels of participants when they were healthy. Lastly, while global *P*-values were assessed previously on these genes [17], we cannot completely rule out the possibility of false positive findings in this study due to multiple comparisons. 

In conclusion, common variants in *VDR* and *RXRA* genes were shown to modify associations between RCC risk and consumption frequency of vitamin D and calcium rich foods, and occupational UV exposure. Additional studies with large sample sizes and thorough genomic coverage of *VDR* and *RXRA* pathway genes, sufficiently powered to investigate gene-environment interactions, are needed to confirm these results and to provide further insight into the role of calcium and vitamin D in RCC etiology. 

## Figures and Tables

**Table 1:** General characteristics of genotyped participants.

	Genotyped participants				
Variables	Cases		Controls		

	N	%	N	%	^† ^ *P*-value

Participants	777	42.9	1,035	57.1	
Sex					
Males	472	60.7	648	62.6	
Females	305	39.3	387	37.4	.42
Age at Interview					
<45	60	7.7	83	8.0	
45–54	197	25.4	287	27.7	
55–64	243	31.3	309	29.9	
65–74	242	31.1	318	30.7	
75+	35	4.5	38	3.7	.30

Mean age (std)	59.5 years (10.4)		59.0 years (10.2)		

Center					
Romania-Bucharest	68	8.8	94	9.1	
Poland-Lodz	80	10.3	189	18.3	
Russia-Moscow	242	31.1	313	30.2	
*Czech Republic	387	49.8	439	42.4	<.001
BMI at Interview					
<25	222	28.6	375	36.3	
25–29.9	330	42.5	432	41.9	
30+	225	29.0	225	21.8	<.001
Tobacco status					
Never	359	46.4	420	40.7	
Ever	415	53.6	613	59.3	.02
Hypertension					
No	434	55.9	638	61.7	
Yes	342	44.1	396	38.3	.01
Familial history of cancer Among 1st degree relatives					
No	512	65.9	745	72.0	
Yes	265	34.1	290	28.0	.01

* Brno, Olomouc, Prague, Ceske-Budejovice; ^†^Crude *P*-values.

**Table 2:** RCC risk by dietary intake frequency of vitamin D and calcium rich foods among genotyped participants.

	Cases		Controls		Adjusted				
Intake Frequency	N	%	N	%	OR	UCI	—	LCI	*P*-trend

Liver									
Low (<33%)	318	41.0	411	39.7	1.00				
Medium (33–66%)	315	40.6	380	36.7	1.11	0.89	—	1.38	
High (>66%)	143	18.4	244	23.6	0.82	0.63	—	1.07	.28
Egg									
Low (<33%)	274	35.3	345	33.3	1.00				
Medium (33–66%)	392	50.5	454	43.9	1.11	0.90	—	1.38	
High (>66%)	110	14.2	236	22.8	0.64	0.48	—	0.86	**.02**
Fresh Water Fish									
Low (<33%)	260	33.5	345	33.3	1.00				
Medium (33–66%)	274	35.3	344	33.2	1.06	0.84	—	1.34	
High (>66%)	242	31.2	346	33.4	0.94	0.74	—	1.19	.60
Salt Water Fish									
Low (<33%)	259	33.4	346	33.4	1.00				
Medium (33–66%)	334	43.0	419	40.5	1.02	0.82	—	1.27	
High (>66%)	183	23.6	270	26.1	0.91	0.71	—	1.18	.53
Total Fish									
Low (<33%)	290	37.4	380	36.7	1.00				
Medium (33–66%)	236	30.4	309	29.9	0.98	0.77	—	1.23	
High (>66%)	250	32.2	346	33.4	0.95	0.75	—	1.19	.65
^^^Total Vitamin D									
Low (<33%)	284	36.6	345	33.3	1.00				
Medium (33–66%)	279	36.0	353	34.1	0.95	0.76	—	1.20	
High (>66%)	213	27.4	337	32.6	0.81	0.63	—	1.03	.09
Cheese									
Low (<33%)	193	24.9	286	27.6	1.00				
Medium (33–66%)	302	38.9	337	32.6	1.31	1.03	—	1.68	
High (>66%)	281	36.2	412	39.8	0.99	0.77	—	1.26	.71
Yogurt									
Low (<33%)	202	26.0	347	33.5	1.00				
Medium (33–66%)	278	35.8	313	30.2	1.48	1.16	—	1.88	
High (>66%)	296	38.1	375	36.2	1.31	1.03	—	1.67	**.04**
Milk									
Low (<33%)	179	23.1	264	25.5	1.00				
Medium (33–66%)	292	37.6	389	37.6	1.10	0.86	—	1.42	
High (>66%)	305	39.3	382	36.9	1.14	0.89	—	1.46	.31
*Total Calcium									
Low (<33%)	226	29.1	345	33.3	1.00				
Medium (33–66%)	274	35.3	355	34.3	1.16	0.91	—	1.48	
High (>66%)	276	35.6	335	32.4	1.20	0.92	—	1.58	.18

All values adjusted for sex, age, study center, self-reported hypertensive status, BMI, and smoking status.

Frequency of dietary intake variables categorized into tertiles based on intake among genotypes controls.

^^^Total Vitamin D (L, M, H) variable based on liver, egg, and salt and fresh water fish consumption.

^^^Also adjusted for years of occupational UV exposure.

* Total calcium variable based on cheese, yogurt, and milk consumption.

**Table 3:** *RXRA* SNPs modify associations between dietary intake frequency of vitamin D rich foods and risk of renal cell carcinoma.

	Genotype association			^§^Dietary intake of egg								^†^Dietary intake of vitamin D rich foods							
						Wild type			≥1 variant					Wild type			≥1 variant		

	Cases/Controls				Cases/Controls			Cases/Controls					Cases/Controls			Cases/Controls			

SNP	N/N	OR	(LCI-UCI)	Egg	N/N	OR	(LCI-UCI)	N/N	OR	(LCI-UCI)	*LRT	Vitamin D	N/N	OR	(LCI-UCI)	N/N	OR	(LCI-UCI)	*LRT

rs1007971 (*7453G > *C*)				Dietary intake of egg								Dietary intake of vitamin D rich foods							

CG/CC	293/338	1.00		Low (<33%)	153/231	1.00		119/107	1.00			Low (<33%)	171/234	1.00		113/111	1.00		
GG	484/697	0.79	(0.64–0.96)	Medium (33–66%)	251/328	1.27	(0.95–1.69)	143/133	1.05	(0.71–1.54)		Medium (33–66%)	164/239	0.90	(0.67–1.20)	115/114	1.04	(0.70–1.55)	
				High (>66%)	79/138	0.99	(0.69–1.43)	31/98	0.32	(0.19–0.53)		High (>66%)	148/224	0.90	(0.66–1.25)	65/113	0.63	(0.39–1.00)	
^^^ * P*-*trend *			*.05*	*P*-*trend *			* .82*			< ***.001***	**.01**	*P*-*trend *			*.53*			*.07*	0.11

rs3118523 (*7060A > *G*)				Dietary intake of Egg								Dietary intake of vitamin D rich foods							

AG/GG	295/321	1.00		Low (<33%)	149/237	1.00		123/101	1.00			Low (<33%)	174/233	1.00		110/112	1.00		
AA	482/714	0.72	(0.58–0.88)	Medium (33–66%)	253/331	1.34	(1.01–1.80)	141/130	0.95	(0.64–1.40)		Medium (33–66%)	165/246	0.87	(0.65–1.16)	114/107	1.13	(0.75–1.68)	
				High (>66%)	79/146	0.98	(0.68–1.42)	31/90	0.29	(0.17–0.49)		High (>66%)	142/235	0.78	(0.57–1.08)	71/102	0.86	(0.54–1.38)	
^^^ * P*-*trend *			*.01*	*P*-*trend *			* .36*			< ***.001***	**.01**	*P*-*trend *			*.14*			*.58*	.59

rs748964 (*5628C > *G*)				Dietary intake of egg								Dietary intake of vitamin D rich foods							

CG/GG	214/224	1.00		Low (<33%)	180/270	1.00		92/68	1.00			Low (<33%)	207/277	1.00		77/68	1.00		
CC	563/811	0.70	(056–0.88)	Medium (33–66%)	291/367	1.26	(0.97–1.64)	103/94	0.91	(0.56–1.46)		Medium (33–66%)	193/263	0.97	(0.74–1.27)	86/90	0.83	(0.52–1.35)	
				High (>66%)	91/174	0.86	(0.62–1.20)	19/62	0.21	(0.11–0.42)		High (>66%)	162/271	0.82	(0.61–1.11)	51/66	0.69	(0.39–1.24)	
^^^ *P*-*trend *			*.01*	*P*-*trend *			* .73*			< ***.001***	**.01**	*P*-*trend *			*.21*			*.21*	.72

rs3118536 (IVS4-542C > A)				Dietary intake of egg								Dietary intake of vitamin D rich foods							

AC/AA	259/310	1.00		Low (<33%)	175/250	1.00		97/88	1.00			Low (<33%)	195/248	1.00		89/97	1.00		
CC	518/725	0.82	(0.67–1.00)	Medium (33–66%)	263/320	1.25	(0.95–1.65)	131/141	0.90	(0.59–1.37)		Medium (33–66%)	168/235	0.91	(0.68–1.21)	111/118	0.96	(0.64–1.44)	
				High (>66%)	79/155	0.81	(0.57–1.16)	31/81	0.35	(0.20–0.60)		High (>66%)	154/242	0.86	(0.63–1.17)	59/95	0.65	(0.40–1.06)	
^^^ *P*-*trend *			*.02*	*P*-*trend *			* .49*			< ***.001***	.21	*P*-*trend *			*.33*			*.10*	.99

rs10776909 (IVS1-4732C > T)				Dietary intake of egg								Dietary intake of vitamin D rich foods							

CT/TT	302/372	1.00		Low (<33%)	159/227	1.00		113/111	1.00			Low (<33%)	174/226	1.00		110/119	1.00		
CC	475/663	0.86	(0.70–1.04)	Medium (33–66%)	242/291	1.24	(0.93–1.67)	152/170	1.00	(0.69–1.46)		Medium (33–66%)	155/205	1.00	(0.74–1.36)	124/148	0.84	(0.58–1.21)	
				High (>66%)	73/145	0.78	(0.54–1.13)	37/91	0.44	(0.26–0.72)		High (>66%)	145/232	0.88	( 0.63–1.22)	68/105	0.65	(0.41–1.02)	
^^^ *P*-*trend *			*.05*	*P*-*trend *			*.37*			***.004***	.37	*P*-*trend *			*.45*			*.06*	.82

Main effects adjusted for age, sex, study center, and smoking status.

Frequency of dietary intake variables categorized into tertiles based on intake among genotyped controls.

^§ ^Dietary effect adjusted for age, study center, BMI, self-reported hypertensive status, and smoking status.

^† ^Dietary effect adjusted for age, study center, BMI, self-reported hypertensive status, smoking status, and occupational UV exposure.

^^^ *P*-trend based on additive model. * Likelihood Ratio Test.

**Table 4:** Effect of *RXRA* SNPs on dietary intake frequency of calcium rich foods and risk of renal cell carcinoma.

	rs3118538 (IVS4-542C > A)							rs10776909 (IVS1-4732C > T)						
	Wild type			≥1 variant				Wild type			≥1 variant			

	Cases/Controls			Cases/Controls				Cases/Controls			Cases/Controls			

	N/N	OR	(UCI-LCI)	N/N	OR	(UCI-LCI)	*LRT	N/N	OR	(UCI-LCI)	N/N	OR	(UCI-LCI)	*LRT

Dietary Intake of Yogurt														
Low (<33%)	133/266	1.00		77/91	1.00			123/247	1.00		87/110	1.00		
Medium (33–66%)	185/212	1.88	(1.34–2.64)	85/91	1.23	(0.73–2.08)		169/198	1.84	(1.29–2.63)	101/105	1.29	(0.81–2.07)	
High (>66%)	199/247	1.69	(1.18–2.42)	97/128	0.87	(0.51–1.50)		182/218	1.71	(1.18–2.49)	114/157	0.94	(0.58–1.54)	
*P*-*trend *			*.01*			*.41*				*.01*			*.58*	
							.09							.**04**

Dietary Intake of Calcium Rich Foods														
Low (<33%)	146/248	1.00		80/97	1.00			135/236	1.00		91/109	1.00		
Medium (33–66%)	180/261	1.18	(0.88–1.59)	94/94	1.22	(0.78–1.89)		162/238	1.21	(0.89–1.66)	112/117	1.10	(0.73–1.64)	
High (>66%)	191/216	1.39	(1.00–1.93)	85/119	0.76	(0.47–1.22)		177/189	1.49	(1.06–2.11)	99/146	0.73	(0.47–1.14)	
*P*-*trend *			*.05*			*.25*				*.02*			*.16*	
							.**03**							.**02**

Dietary effect adjusted for age, sex, study center, BMI, self-reported hypertensive status, and smoking status.

Frequency of dietary intake variables categorized into tertiles based on intake among genotyped controls.

Calcium rich foods based on consumption of cheese, yogurt, and milk.

Main effects adjusted for age, sex, study center, and smoking status.

rs3118538 (IVS4-542C > A) main effects: AC (0.68(0.39–1.18)), AA (0.58(0.34–0.99)), *P*-trend = .02; AC/AA (0.82(0.67–1.00)).

rs10776909 (IVS1-4732C > T) main effects: CT (0.69(0.42–1.13)), TT (0.62(0.38–1.00)), *P*-trend = .05; CT/TT (0.86(0.70–1.04)).

*Likelihood Ratio Test.

**Table 5:** *VDR* haplotypes and RCC risk among males before and after stratification by cumulative.

						Low cumulative exposure					Medium cumulative exposure					High cumulative exposure					
	Male Participants					< **6.00** (low-exposure-unit-years)					**6.00**–**16.10** (low-exposure-unit-years)					>**16.10** (low-exposure-unit-years)					

*VDR*	Cases	Controls	$\hat{\;\;}$ Main effect			Cases	Controls				Cases	Controls				Cases	Controls				

Haplotypes	%	%	OR	(LCI-UCI)	*P*-value	%	%	OR	(LCI-UCI)	*P*-value	%	%	OR	(LCI-UCI)	*P*-value	%	%	OR	(LCI-UCI)	*P*-value	*LRT

5′-rs2254210, rs2853564, rs4760648-3′																					
G-A-T	39.9	43.8	1.00			44.2	41.5	1.00			38.5	42.7	1.00			37.4	46.2	1.00			
A-G-C	31.7	28.3	1.27	(1.03–1.57)	**.02**	29.7	31.0	0.93	(0.63–1.37)	.72	31.7	28.7	1.23	(0.87–1.74)	.25	33.5	25.6	1.70	(1.14–2.53)	**.01**	
G-A-C	19.5	18.5	1.19	(0.94–1.51)	.16	16.9	18.0	0.84	(0.52–1.37)	.49	22.2	17.2	1.46	(0.99–2.16)	.06	19.2	20.3	1.15	(0.75–1.78)	.52	
G-G-C	5.7	6.6	0.97	(0.67–1.42)	.88	5.3	7.1	0.74	(0.36–1.50)	.40	5.5	7.7	0.76	(0.42–1.39)	.38	6.6	5.3	1.83	(0.88–3.81)	.11	
*Global P*					*.31*					*.82*					*.21*					*.13*	
																					.06

$\hat{\;\;}$ Main effects adjusted for age, study center, BMI, self-reported hypertensive status, and smoking status.

Occupational UV effect adjusted for age, study center, BMI, self-reported hypertensive status, smoking status, and dietary intake frequency of vitamin D rich foods.

rs2245210 (IVS3-816G > A) main effects: *AG* (0.86(0.63–1.18)), *GG* (0.71(0.52–0.98)), *P*-trend = .02;

rs2853564 (IVS2-1930A > G) main effects: *AG* (1.00(0.75–1.33)), *GG* (0.77(0.58–1.04)), *P*-trend = .03;

rs4760648 (IVS2-4108C > T) main effects: *CT* (0.81(0.65–1.00)), *TT* (0.64(0.48–0.85)), *P*-trend = .002.

* Likelihood Ratio Test.
